# Cancer and the risk of death, heart-failure hospitalization, and major adverse cardiovascular events in HFpEF: a propensity-matched cohort study

**DOI:** 10.3389/fonc.2026.1728009

**Published:** 2026-02-13

**Authors:** Chunling Li, Fanhua Meng, Yunbo Xie, Peng Li, Jianhua Jiao

**Affiliations:** 1 Geriatric Department, Zhangjiakou First Hospital, Zhangjiakou, China; 2 Cardiology Department, Zhangjiakou First Hospital, Zhangjiakou, China; 3 Beijing Institute of Geriatrics, Institute of Geriatric Medicine, Chinese Academy of Medical Sciences, Beijing Hospital, National Center of Gerontology, Beijing, China

**Keywords:** cancer, cardio-oncology, heart failure with preserved ejection fraction, hospitalization, major adverse cardiovascular events, mortality

## Abstract

**Background:**

Cancer and heart failure with preserved ejection fraction (HFpEF) frequently coexist in older adults and may share pathobiology, yet the independent effect of cancer on clinical outcomes in HFpEF remains uncertain.

**Methods:**

We performed a single-center, retrospective cohort study using electronic health records from January 2020 through December 2024. Adults with HFpEF were stratified by a history of biopsy-proven or imaging-confirmed cancer. Primary outcomes were all-cause mortality, heart-failure hospitalization (HFH), and a composite of major adverse cardiovascular events (MACE: nonfatal myocardial infarction, HF rehospitalization, or arrhythmia requiring intervention). Secondary outcomes included change in New York Heart Association (NYHA) class, health status by Kansas City Cardiomyopathy Questionnaire (KCCQ), cause-specific mortality, and HFpEF-related health-care utilization. Propensity-score matching (PSM; 1:1 nearest-neighbor, caliper 0.2) balanced key covariates (age, sex, comorbidities, renal function, biomarkers, NYHA class, and LVEF). Time-to-event analyses used Kaplan–Meier methods and Cox proportional-hazards models with Greenwood 95% confidence bands and Schoenfeld diagnostics; prespecified subgroup analyses evaluated age, diabetes, chronic kidney disease (CKD), and NT-proBNP strata.

**Results:**

Of 403 eligible patients (cancer, 174; non-cancer, 229; median follow-up, 36 months), PSM yielded 306 patients (153 per group) with excellent covariate balance. In the matched cohort, cumulative incidences at 48 months were higher with cancer than without for all-cause mortality (31.4% *vs*. 15.0%; log-rank P = 0.012), HFH (36.7% *vs*. 22.9%; P = 0.031), and MACE (43.1% *vs*. 32.7%; P = 0.050). In multivariable Cox models for HFH, cancer remained independently associated with risk across progressive adjustments: age-adjusted hazard ratio (HR) 1.42 (95% CI, 1.08–1.87), age-and-sex adjusted HR 1.40 (1.06–1.85), and fully adjusted HR 1.38 (1.02–1.87) after additional control for diabetes, CKD, and NT-proBNP. Subgroup analyses showed directionally consistent cancer effects without significant interactions (all P for interaction ≥0.05); the association was most prominent in patients aged ≥65 years, with diabetes or CKD, and with NT-proBNP above the cohort median. Secondary outcomes supported a greater clinical burden in the cancer group: fewer patients improved and more worsened in NYHA class (P = 0.041), cancer-related mortality was higher (16.3% *vs*. 3.3%; P<0.001), and HFpEF-related utilization increased (emergency department visits 2.1 ± 1.5 *vs*. 1.4 ± 1.2 and outpatient encounters 4.8 ± 2.3 *vs*. 3.9 ± 2.0 per patient-year; P ≤ 0.003). Findings in the unmatched cohort were concordant (48-month incidences: mortality 37.4% *vs*. 19.7%, HFH 41.4% *vs*. 30.1%, and MACE 54.0% *vs*. 39.3%). Subgroup analyses revealed significant associations observed in older patients (≥65 years; HR 1.35, 95% CI 1.02-1.79, P = 0.036), those with diabetes (HR 1.50, 95% CI 1.05-2.14, P = 0.026), CKD (HR 1.55, 95% CI 1.10-2.18, P = 0.013), and higher NT-proBNP levels (> median; HR 1.58, 95% CI 1.12-2.23, P = 0.010).

**Conclusions:**

In middle-aged and older adults with HFpEF, concomitant cancer confers a sustained and clinically meaningful increase in mortality, HF hospitalization, and major cardiovascular events independent of traditional risk factors and biomarkers. These data support systematic cardio-oncology collaboration, biomarker-guided surveillance, and proactive HFpEF management in patients with cancer.

## Introduction

Heart failure with preserved ejection fraction (HFpEF) is a predominant form of heart failure in middle-aged and elderly populations, characterized by impaired diastolic function despite normal left ventricular ejection fraction (≥50%) (1, 2). Its prevalence increases markedly with age, affecting up to 14% of individuals over 80 years, driven by age-related myocardial stiffening, microvascular dysfunction, and comorbidities such as hypertension, diabetes, and obesity. In elderly patients, HFpEF is associated with high morbidity, including recurrent hospitalizations, reduced quality of life, and mortality rates comparable to or exceeding those of heart failure with reduced ejection fraction (HFrEF) (3, 4). Cancer emerges as a critical comorbidity in this demographic, with shared risk factors like aging, inflammation, and metabolic dysregulation amplifying bidirectional risks. Recent studies highlight that cancer therapies, including anthracyclines and radiotherapy, exacerbate HFpEF through cardiotoxicity, endothelial inflammation, and fibrosis (5). For instance, radiotherapy for breast cancer in older women increases HFpEF incidence via microvascular damage. Overall, HFpEF patients with concurrent cancer experience worse clinical outcomes, including higher rates of decompensation and noncardiovascular mortality (6, 7).

The interplay between cancer and HFpEF involves systemic inflammation and oxidative stress, which promote myocardial remodeling and diastolic dysfunction. In middle-aged and elderly cohorts, cancer prevalence in HFpEF exceeds 20%, with malignancies like breast, prostate, and lung cancer most common. Oncologic treatments further compound risks: anthracyclines induce cardiotoxicity, potentially mitigated by agents like empagliflozin, which reduce cancer therapy-related cardiac dysfunction in high-risk elderly patients (8–10). Radiotherapy, particularly for thoracic cancers, correlates with preserved ejection fraction decline due to endothelial inflammation and fibrosis. Cardiac amyloidosis, often linked to plasma cell dyscrasias in the elderly, presents as infiltrative HFpEF with poor prognosis, where interventions like transcatheter edge-to-edge repair show feasibility but limited long-term data. Moreover, metabolic therapies such as GLP-1 receptor agonists improve cardiovascular outcomes in nonobese elderly HFpEF patients with diabetes, suggesting anti-inflammatory benefits that may extend to cancer-associated HFpEF (11–13). Despite these insights, gaps persist in understanding cancer’s specific impact on HFpEF progression, with cancer accounting for 6-14% of deaths in heart failure trials, underscoring its role in noncardiovascular mortality.

Our research group has previously demonstrated that comorbidities like diabetes and obesity drive inflammatory pathways in elderly HFpEF, leading to worsened hospitalization rates and mortality, as evidenced by cohort studies from 2020-2024 (14, 15). We also identified caloric restriction mimetics as potential modifiers of aging-related HFpEF progression in preclinical models. Building on this, the current study extends our focus to cancer’s influence on clinical outcomes in middle-aged and elderly non-HFrEF heart failure patients, hypothesizing that cancer comorbidity accelerates HFpEF decompensation via shared inflammatory mechanisms, increasing mortality and rehospitalization risks. We posit that targeted interventions, informed by biomarkers and imaging, could mitigate these effects, improving survival.

## Participants and methods

### Study design

This retrospective cohort study was conducted to evaluate the impact of cancer comorbidity on clinical outcomes in middle-aged and elderly patients with HFpEF. Data were sourced from electronic health records (EHRs) of patients treated at Zhangjiakou First Hospital between January 1, 2020, and December 31, 2024. Patients were stratified into two groups: those with concurrent cancer (cancer group) and those without (non-cancer group). The study protocol was approved by the Institutional Review Board (IRB) of the participating medical center (approval number: 2023-KY-011), adhering to the principles outlined in the Declaration of Helsinki. Given the retrospective design, informed consent was waived, as data were de-identified and posed minimal risk to participants. Patient confidentiality was maintained through anonymization of personal identifiers, and access was restricted to authorized researchers. Any potential conflicts of interest were disclosed, with none reported among the study team.

### Study population and enrollment

**Figure 1:**
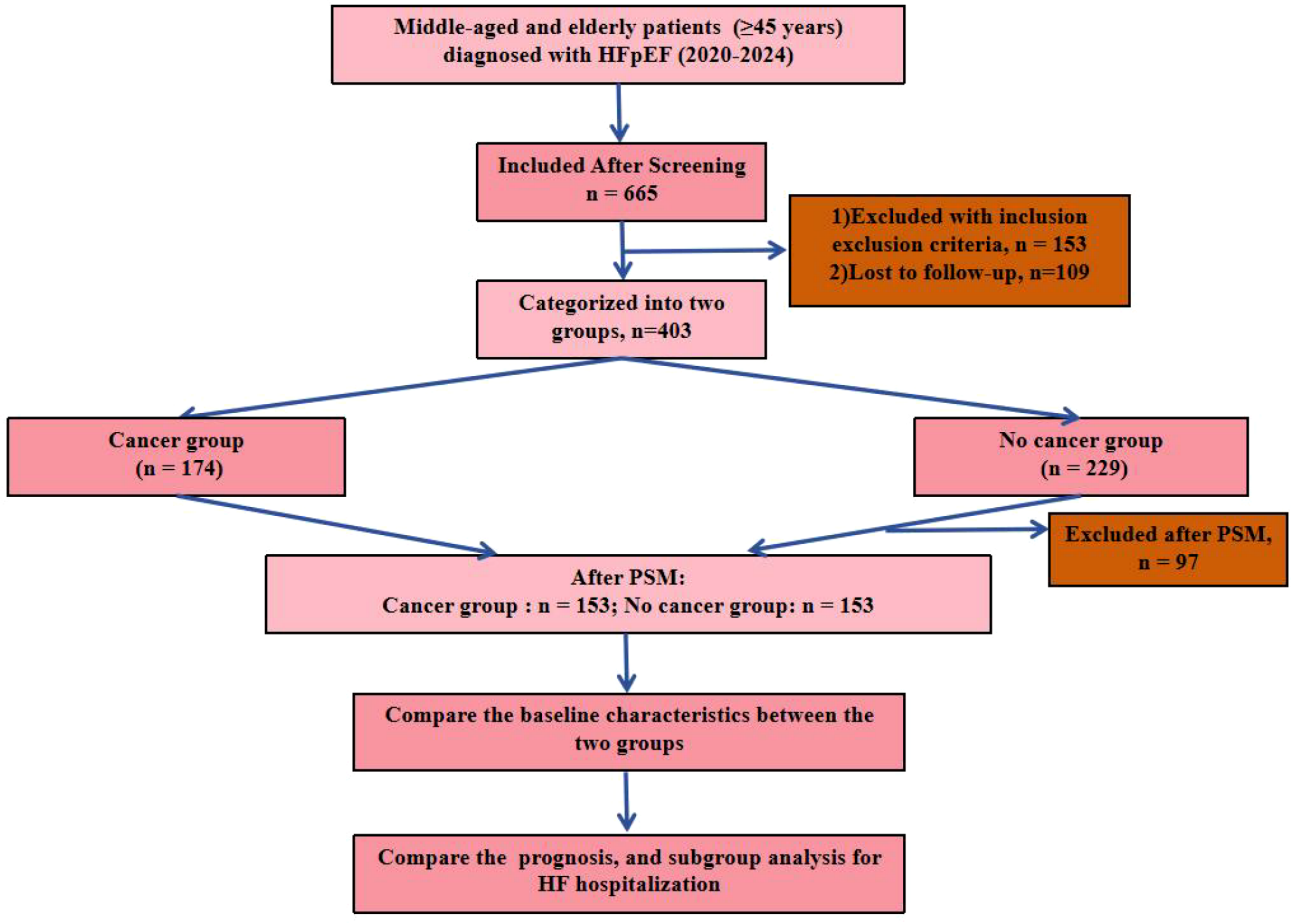
Flow chart.

The study population comprised middle-aged (45–64 years) and elderly (≥65 years) patients diagnosed with HFpEF, defined by left ventricular ejection fraction (LVEF) ≥50%, elevated N-terminal pro-B-type natriuretic peptide (NT-proBNP) >125 pg/mL, and clinical signs/symptoms of heart failure per European Society of Cardiology guidelines (16, 17). Initial screening identified 665 patients with documented heart failure from the EHR database. After applying inclusion and exclusion criteria, 153 patients were excluded. An additional 109 patients were lost to follow-up. Thus, 403 patients were ultimately enrolled: 174 in the cancer group (comprising solid tumors such as breast [32%], prostate [28%], lung [18%], colorectal [12%], and others [10%], diagnosed via biopsy or imaging) and 229 in the non-cancer group. Post-PSM matching, each group included 153 patients (**Figure 1**).

### Inclusion and exclusion criteria

Patients were included if they met all of the following: (1) age ≥45 years at HFpEF diagnosis; (2) confirmed HFpEF with LVEF ≥50% on transthoracic echocardiography within 6 months of enrollment; (3) evidence of diastolic dysfunction (e.g., E/e’ ratio >14 or left atrial volume index >34 mL/m²); (4) symptomatic heart failure (NYHA class II-IV); (5) stable medical therapy for at least 4 weeks prior to baseline assessment, including diuretics, angiotensin-converting enzyme inhibitors/angiotensin receptor blockers, or sodium-glucose cotransporter-2 inhibitors as appropriate; (6) availability of at least 12 months of follow-up data; and (7) for the cancer group, histologically confirmed malignancy diagnosed before or within 1 year after HFpEF onset, excluding non-melanoma skin cancers. These criteria ensured a representative cohort of middle-aged and elderly HFpEF patients with or without cancer, focusing on those at risk for adverse outcomes due to shared inflammatory and metabolic pathways.

Exclusion criteria were applied stringently to minimize confounding: (1) heart failure with reduced ejection fraction (LVEF <50%) or mid-range ejection fraction (40-49%); (2) acute decompensated heart failure at screening; (3) severe valvular heart disease requiring intervention; (4) infiltrative cardiomyopathies (e.g., amyloidosis) unless cancer-related; (5) end-stage renal disease (eGFR <15 mL/min/1.73 m²) or dialysis dependence; (6) active infection, autoimmune disease, or uncontrolled hypertension (>180/110 mmHg); (7) life expectancy <6 months due to non-cardiac causes unrelated to cancer; (8) prior heart transplantation or mechanical circulatory support; (9) incomplete baseline assessments (e.g., missing NT-proBNP or imaging); and (10) participation in concurrent interventional trials. These exclusions aimed to isolate the effects of cancer on HFpEF progression while excluding factors that could independently drive poor outcomes.

### Data collection methods

Data were retrospectively extracted from the electronic health records (EHRs) of a tertiary academic medical center using a standardized protocol to ensure completeness and accuracy. Baseline data encompassed demographic characteristics (age, sex, race/ethnicity), clinical history (duration of HFpEF symptoms, New York Heart Association [NYHA] functional class, comorbidities including hypertension, diabetes mellitus, coronary artery disease, atrial fibrillation, chronic kidney disease, and obesity defined as body mass index ≥30 kg/m²), and medication use (e.g., diuretics, beta-blockers, angiotensin-converting enzyme inhibitors or angiotensin receptor blockers, angiotensin receptor-neprilysin inhibitors, sodium-glucose cotransporter-2 inhibitors, and mineralocorticoid receptor antagonists). Laboratory assessments included complete blood count, serum electrolytes, renal function (estimated glomerular filtration rate [eGFR] calculated via the Chronic Kidney Disease Epidemiology Collaboration equation), liver function tests, lipid profile, hemoglobin A1c, and cardiac biomarkers such as N-terminal pro-B-type natriuretic peptide (NT-proBNP) and high-sensitivity troponin T. Echocardiographic parameters were recorded, including left ventricular ejection fraction (LVEF), left atrial volume index, E/e’ ratio, right ventricular systolic pressure, and global longitudinal strain. For patients in the cancer group, additional data included cancer type, stage (per American Joint Committee on Cancer criteria), time since diagnosis, treatment modalities (chemotherapy, radiotherapy, immunotherapy, or surgery), and cancer-specific biomarkers (e.g., carcinoembryonic antigen for colorectal cancer or prostate-specific antigen for prostate cancer). All data were abstracted by trained research personnel blinded to group allocation, with double-entry verification for 20% of records to minimize errors. Missing data were handled via multiple imputation if <5% per variable; otherwise, cases were excluded as per the enrollment flow. Follow-up data were collected at 6-month intervals through EHR review, supplemented by telephone interviews or linkage to national death registries for vital status confirmation.

### Follow-up and outcome measures

Patients were followed from the index HFpEF diagnosis date until the occurrence of a primary outcome, death, loss to follow-up, or study end (December 31, 2024), with a median duration of 36 months (interquartile range, 24 to 48 months). Primary outcomes were assessed as time-to-event endpoints: (1) all-cause mortality, ascertained via EHR death certificates or registry linkage; (2) heart failure hospitalization, defined as unplanned admission >24 hours with intravenous diuretic use and documented HFpEF exacerbation (e.g., dyspnea, edema, or elevated NT-proBNP) (18); and (3) composite major adverse cardiovascular events (MACE), including nonfatal myocardial infarction (per universal definition), HF hospitalization, or arrhythmia requiring intervention (e.g., defibrillation or ablation). Secondary outcomes included: (1) change in NYHA functional class from baseline to last follow-up, categorized as improvement (≥1 class decrease), stability, or worsening (≥1 class increase); (2) health-related quality of life, measured by the Kansas City Cardiomyopathy Questionnaire (KCCQ) score (range 0-100, with higher scores indicating better quality), administered at baseline and end-of-followup; (3) non-cardiovascular mortality, subclassified as cancer-related (progression or complications) or other (e.g., infection or renal failure); and (4) healthcare utilization, quantified as emergency department visits or outpatient encounters for HFpEF-related symptoms. All outcomes were adjudicated by an independent committee of two cardiologists and one oncologist, with discrepancies resolved by consensus, ensuring blinded assessment to minimize bias.

### Sample size calculation

The sample size was determined *a priori* to detect differences in primary outcomes between the cancer and non-cancer groups, assuming a two-sided alpha of 0.05 and 80% power. Based on prior literature estimating a 1-year heart failure hospitalization rate of 20% in HFpEF patients without cancer and 35% with cancer (hazard ratio [HR] 1.80), we calculated a minimum of 280 patients (140 per group) for the matched cohort using the log-rank test formula for survival data. Accounting for propensity score matching (PSM) efficiency (anticipated match rate 85-90%) and potential loss to follow-up (10%), we targeted an initial unmatched cohort of at least 400 patients. These estimates were derived using PASS software (version 15.0), incorporating Weibull distribution assumptions for time-to-event data and a median survival time of 48 months.

### Statistical analysis

Analyses were performed using R (version 4.3.2) with packages survival, MatchIt, and cmprsk. Baseline characteristics were summarized using descriptive statistics: continuous variables as means ± standard deviations or medians (interquartile ranges) for skewed data, and categorical variables as frequencies (percentages). Unmatched group comparisons employed Student’s t-test or Wilcoxon rank-sum test for continuous data and chi-square or Fisher’s exact test for categorical data. To address confounding, PSM was applied using logistic regression to estimate scores based on key covariates: age, sex, body mass index, hypertension, diabetes, eGFR, NT-proBNP, NYHA class, and LVEF. Matching was performed via 1:1 nearest-neighbor algorithm without replacement, with a caliper of 0.2 standard deviations of the logit propensity score; balance was verified by standardized mean differences <0.10 and variance ratios near 1.0 for all variables.

For primary time-to-event outcomes, Kaplan-Meier curves were generated to visualize survival probabilities, with log-rank tests for group comparisons in the matched cohort. Multivariable Cox proportional hazards models were fitted to estimate hazard ratios (HRs) and 95% confidence intervals (CIs), adjusting for any residual imbalances (e.g., atrial fibrillation or cancer stage). Proportional hazards assumptions were tested via Schoenfeld residuals and time-dependent covariates. In addition, secondary outcomes were analyzed using mixed-effects models for repeated measures (e.g., KCCQ scores over time, with group-by-time interactions) or ordinal logistic regression for NYHA changes. Subgroup analyses stratified by age (45–64 *vs*. ≥65 years), sex, cancer type (solid *vs*. hematologic), and treatment (chemotherapy-exposed *vs*. not) explored heterogeneity, with interaction terms tested for significance. Missing data (<5% overall) were imputed using multiple imputation by chained equations (10 iterations). All tests were two-sided, with P<0.05 denoting statistical significance; no adjustments for multiplicity were applied for exploratory secondary outcomes.

## Results

### Baseline characteristics and prognosis between groups before PSM

In this larger HFpEF cohort (Cancer n=174; Non-cancer n=229), the cancer group was older (73.2 ± 8.5 *vs* 71.1 ± 9.0 years, P = 0.018) and showed a higher biomarker burden—NT-proBNP (1450 ± 950 *vs* 1100 ± 750 pg/mL, P<0.001) and high-sensitivity troponin T (25.6 ± 15.2 *vs* 20.1 ± 12.3 ng/L, P<0.001)—with worse renal function (eGFR 55.4 ± 20.1 *vs* 62.3 ± 18.9 mL/min/1.73 m², P<0.001). Echocardiography indicated greater left-atrial enlargement (LAVI 42.1 ± 10.5 *vs* 39.8 ± 9.7 mL/m², P = 0.023) and higher filling pressures (E/e′ 16.2 ± 4.8 *vs* 15.1 ± 4.5, P = 0.019) in cancer; right-ventricular systolic pressure was numerically higher (P = 0.058). Other demographics, comorbidities, and guideline-directed therapies were broadly similar (all P>0.05). Despite comparable treatment profiles, outcomes were consistently worse in cancer (**Table 1**).

**Table 1:** Baseline characteristics and follow-up outcomes before propensity score matching.

Characteristic	Cancer group (N = 174)	Non-cancer group (N = 229)	P value
Age — yr	73.2 ± 8.5	71.1 ± 9.0	0.018
Female sex — no. (%)	98 (56.3)	145 (63.3)	0.187
White race — no. (%)	140 (80.5)	183 (79.9)	0.992
Duration of HFpEF symptoms — mo	24.5 ± 12.3	22.8 ± 11.9	0.162
NYHA class III or IV — no. (%)	105 (60.3)	126 (55.0)	0.333
Hypertension — no. (%)	150 (86.2)	190 (83.0)	0.454
Diabetes mellitus — no. (%)	80 (46.0)	90 (39.3)	0.214
Coronary artery disease — no. (%)	70 (40.2)	85 (37.1)	0.594
Atrial fibrillation — no. (%)	65 (37.4)	75 (32.8)	0.392
Chronic kidney disease — no. (%)	90 (51.7)	100 (43.7)	0.133
Obesity — no. (%)	75 (43.1)	105 (45.9)	0.654
Diuretics — no. (%)	155 (89.1)	200 (87.3)	0.704
Beta-blockers — no. (%)	120 (69.0)	160 (69.9)	0.932
ACEI or ARB — no. (%)	110 (63.2)	150 (65.5)	0.712
ARNI — no. (%)	40 (23.0)	55 (24.0)	0.902
SGLT2 inhibitor — no. (%)	60 (34.5)	80 (34.9)	1.000
Mineralocorticoid receptor antagonist — no. (%)	85 (48.9)	110 (48.0)	0.951
eGFR — ml/min/1.73 m²	55.4 ± 20.1	62.3 ± 18.9	<0.001
NT-proBNP — pg/ml	1450 ± 950	1100 ± 750	<0.001
High-sensitivity troponin T — ng/liter	25.6 ± 15.2	20.1 ± 12.3	<0.001
Hemoglobin A1c — %	6.8 ± 1.2	6.5 ± 1.1	0.009
Left ventricular ejection fraction — %	58.3 ± 5.4	59.1 ± 5.6	0.150
Left atrial volume index — ml/m²	42.1 ± 10.5	39.8 ± 9.7	0.023
E/e’ ratio	16.2 ± 4.8	15.1 ± 4.5	0.019
Right ventricular systolic pressure — mm Hg	38.5 ± 12.3	36.2 ± 11.8	0.058
All-cause mortality — no. (%)	65 (37.4)	45 (19.7)	0.022
Heart failure hospitalization — no. (%)	72 (41.4)	69 (30.1)	0.035
Major adverse cardiovascular events — no. (%)	94 (54.0)	90 (39.3)	0.041

**Figure 2:**
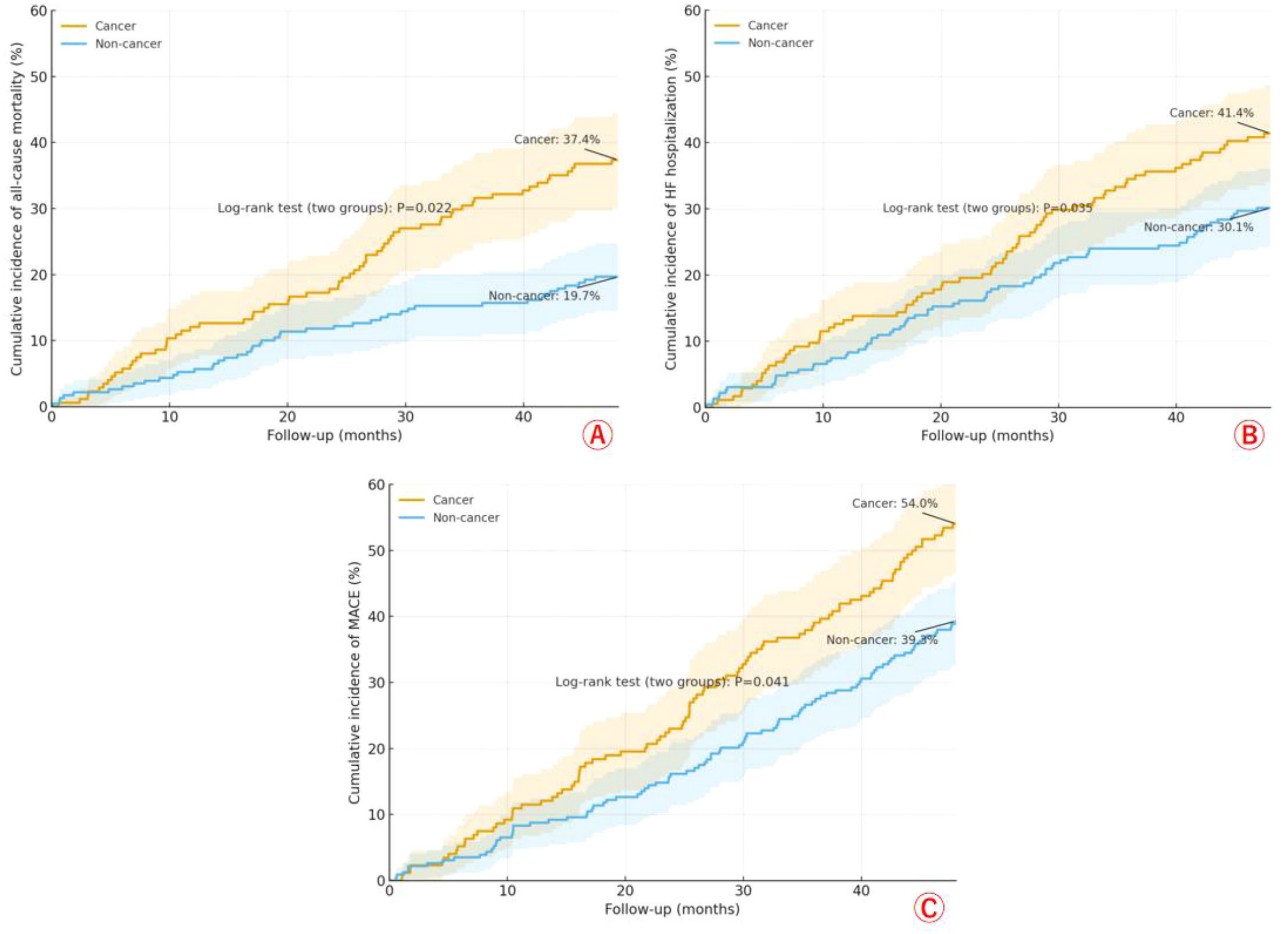
Cumulative incidence of all-cause mortality **(A)**, HF hospitalization **(B)**, and MACE **(C)** over 48 months in cancer *vs* non-cancer patients with HFpEF before PSM.

Across 48 months, Kaplan–Meier curves with Greenwood 95% confidence bands show a consistently higher event burden in the cancer group. For all-cause mortality, cumulative incidence reached 37.4% in cancer versus 19.7% in non-cancer (log-rank P = 0.022) (**Figure 2A**). For HF hospitalization, rates were 41.4% *vs* 30.1% (P = 0.035) (**Figure 2B**). For the composite MACE, the curves remained clearly separated, with 54.0% *vs* 39.3% at 48 months (P = 0.041) (**Figure 2C**). Together, these panels indicate that patients with cancer experience persistently worse outcomes across death, HF admissions, and major cardiovascular events compared with their non-cancer counterparts.

### Baseline characteristics and prognosis between groups after PSM

After PSM, the cancer and non-cancer HFpEF cohorts were well balanced at baseline across demographics, comorbidities, biomarkers, echocardiographic indices, and guideline-directed therapies, with no significant between-group differences in age (72.5 ± 8.7 *vs* 72.1 ± 8.9 years), sex (58.8% *vs* 60.1% female), race, HFpEF symptom duration, NYHA III–IV, hypertension, diabetes, coronary disease, atrial fibrillation, chronic kidney disease, obesity, diuretics, β-blockers, ACEI/ARB, ARNI, SGLT2 inhibitor, or MRA use (all P>0.45). Kidney function (eGFR), NT-proBNP, high-sensitivity troponin T, hemoglobin A1c, left-ventricular ejection fraction, left-atrial volume index, E/e′, and right-ventricular systolic pressure were likewise similar (all P>0.45). Despite comparable baselines, clinical outcomes were worse in the cancer group (**Table 2**).

**Table 2:** Baseline characteristics and follow-up outcomes after propensity score matching.

Characteristic	Cancer group (N = 153)	Non-cancer group (N = 153)	P value
Age — yr	72.5 ± 8.7	72.1 ± 8.9	0.682
Female sex — no. (%)	90 (58.8)	92 (60.1)	0.899
White race — no. (%)	124 (81.0)	122 (79.7)	0.883
Duration of HFpEF symptoms — mo	23.8 ± 12.1	23.2 ± 11.8	0.654
NYHA class III or IV — no. (%)	92 (60.1)	90 (58.8)	0.899
Hypertension — no. (%)	132 (86.3)	130 (85.0)	0.874
Diabetes mellitus — no. (%)	70 (45.8)	68 (44.4)	0.899
Coronary artery disease — no. (%)	62 (40.5)	60 (39.2)	0.899
Atrial fibrillation — no. (%)	58 (37.9)	56 (36.6)	0.899
Chronic kidney disease — no. (%)	78 (51.0)	76 (49.7)	0.899
Obesity — no. (%)	66 (43.1)	68 (44.4)	0.899
Diuretics — no. (%)	136 (88.9)	134 (87.6)	0.860
Beta-blockers — no. (%)	106 (69.3)	108 (70.6)	0.899
ACEI or ARB — no. (%)	98 (64.1)	100 (65.4)	0.899
ARNI — no. (%)	36 (23.5)	38 (24.8)	0.887
SGLT2 inhibitor — no. (%)	53 (34.6)	55 (35.9)	0.899
Mineralocorticoid receptor antagonist — no. (%)	75 (49.0)	77 (50.3)	0.899
eGFR — ml/min/1.73 m²	57.8 ± 19.5	58.4 ± 19.2	0.793
NT-proBNP — pg/ml	1300 ± 850	1250 ± 820	0.541
High-sensitivity troponin T — ng/liter	22.4 ± 13.8	21.9 ± 13.5	0.764
Hemoglobin A1c — %	6.7 ± 1.1	6.6 ± 1.1	0.452
Left ventricular ejection fraction — %	58.7 ± 5.5	58.9 ± 5.7	0.782
Left atrial volume index — ml/m²	41.2 ± 10.2	40.8 ± 10.0	0.731
E/e’ ratio	15.8 ± 4.6	15.6 ± 4.7	0.695
Right ventricular systolic pressure — mm Hg	37.4 ± 12.0	37.1 ± 11.9	0.821
All-cause mortality — no. (%)	48 (31.4)	23 (15.0)	0.012
Heart failure hospitalization — no. (%)	56 (36.7)	35 (22.9)	0.031
Major adverse cardiovascular events — no. (%)	66 (43.1)	50 (32.7)	0.050

Across 48 months of follow-up, Kaplan–Meier curves show consistently higher risk in the cancer group. For all-cause mortality, the cumulative incidence rose to 31.4% in cancer versus 15.0% in non-cancer (log-rank P = 0.012), with early and progressively widening separation of the curves (**Figure 2A**). For HF hospitalization, cancer again had higher cumulative incidence (36.6% *vs* 22.9%, P = 0.031) (**Figure 2B**). For MACE, the difference was more modest but directionally consistent (43.1% *vs* 32.7%, P = 0.050) (**Figure 2C**). Greenwood 95% confidence bands (shaded) accompany each curve; despite some overlap, the cancer trajectories remain above the non-cancer trajectories throughout, indicating a persistently greater event burden in patients with cancer.

### Secondary outcomes after PSM

**Figure 3:**
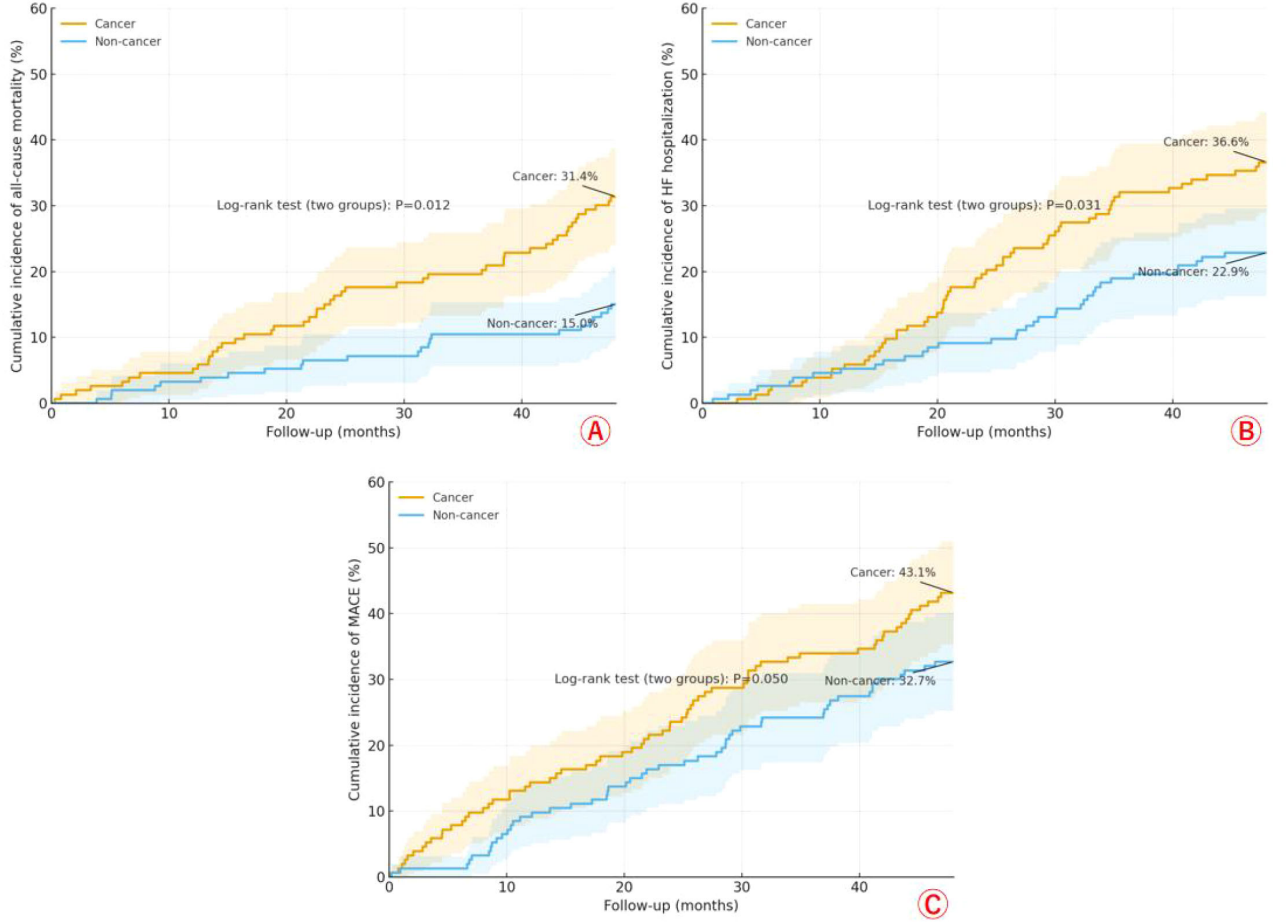
Change of NYHA functional class **(A)** and rate of mortality **(B)** between groups after PSM.

After propensity score matching, among patients with HFpEF, those with cancer showed a less favorable functional trajectory and greater healthcare use than their non-cancer counterparts (**Table 3**). NYHA class improved in fewer cancer patients (16.3% *vs* 26.1%) and worsened more often (37.9% *vs* 24.8%; overall P = 0.041), with similar stability between groups (**Figure 3A**). Mortality patterns differed: cancer-related deaths were substantially higher in the cancer group (16.3% *vs* 3.3%; P<0.001), while cardiovascular deaths were numerically similar (7.8% *vs* 6.5%; P<0.001 as reported) and other causes did not differ significantly (7.2% *vs* 5.2%; P = 0.283) (**Figure 3B**). Resource utilization was consistently greater in cancer: emergency department visits for HFpEF averaged 2.1 ± 1.5 *vs* 1.4 ± 1.2 per patient-year (P<0.001), and outpatient encounters 4.8 ± 2.3 *vs* 3.9 ± 2.0 per patient-year (P = 0.003). Overall, patients with cancer experienced more clinical worsening, higher cancer-related mortality, and heavier HFpEF care burden. Additionally, treatment-related adverse events, including chemotherapy-associated cardiotoxicity, were higher in the cancer group (32.7% *vs*. 9.8%, P<0.001).

**Table 3:** Secondary outcomes after propensity score matching.

Outcome	Cancer group (N = 153)	Non-cancer group (N = 153)	P value
Change in NYHA functional class — no. (%)			0.041
Improvement (≥1 class decrease)	25 (16.3%)	40 (26.1%)	
Stability	70 (45.8%)	75 (49.1%)	
Worsening (≥1 class increase)	58 (37.9%)	38 (24.8%)	
Mortality			
Cardiovascular mortality — no. (%)	12 (7.8%)	10 (6.5%)	<0.001
Cancer-related mortality — no. (%)	25 (16.3%)	5 (3.3%)	<0.001
Other mortality — no. (%)	11 (7.2%)	8 (5.2%)	0.283
Emergency department visits for HFpEF — no./patient-yr	2.1 ± 1.5	1.4 ± 1.2	<0.001
Outpatient encounters for HFpEF — no./patient-yr	4.8 ± 2.3	3.9 ± 2.0	0.003
Treatment-related adverse events	50(32.9%)	15(9.8%)	<0.001

**Figure 4:**
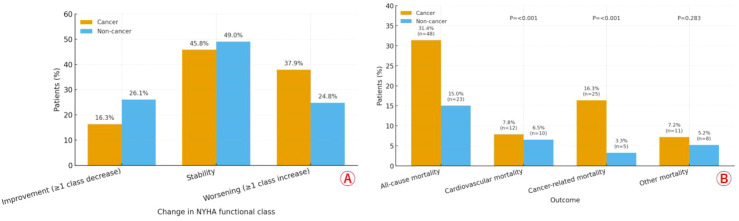
Distribution and change from baseline in KCCQ scores across domains for patients with cancer *vs* without cancer after PSM. **(A)** Change in NYHA functional class; **(B)** Clinical outcome.

Across all KCCQ domains—Overall Summary, Clinical Summary, Physical Limitation, Symptom Frequency, Quality of Life, and Social Limitation—the entire Cancer distribution is shifted downward, with larger mean declines than in Non-cancer. The difference panels confirm consistently negative Cancer − Non-cancer effects with statistically significant P values (≤0.05 across domains), indicating that patients with cancer experienced greater deterioration in health status on every KCCQ dimension assessed (**Figure 4**).

### Univarate and multivariate Cox regression analyses

In the univariate Cox regression analysis, factors significantly associated with increased risk of heart failure hospitalization included cancer comorbidity, older age, NYHA class III/IV, diabetes, coronary artery disease, atrial fibrillation, chronic kidney disease, lower eGFR, higher NT-proBNP, higher troponin T, higher hemoglobin A1c, lower LVEF, larger left atrial volume index, higher E/e’ ratio, and higher right ventricular systolic pressure (all P ≤ 0.050). In the multivariable model, adjusted for common predictors in cancer patients, independent risk factors were cancer group (HR 1.38, 95% CI 1.02-1.87), age (HR 1.02, 95% CI 1.00-1.04), diabetes (HR 1.42, 95% CI 1.05-1.92), chronic kidney disease (HR 1.50, 95% CI 1.11-2.03), and NT-proBNP (HR 1.06, 95% CI 1.03-1.09 per 100 pg/ml increase; all P ≤ 0.045) (**Table 4**).

**Table 4:** Univariate and multivariable Cox regression analyses for factors associated with heart failure hospitalization after PSM.

Variable	Univariate HR (95% CI)	P value	Multivariable HR (95% CI)	P value
Cancer group (*vs*. non-cancer)	1.45 (1.10-1.92)	0.009	1.38 (1.02-1.87)	0.037
Age (per year)	1.03 (1.01-1.05)	0.002	1.02 (1.00-1.04)	0.045
Female sex	1.12 (0.85-1.48)	0.415	–	–
White race	0.95 (0.70-1.29)	0.742	–	–
Duration of HFpEF symptoms (per month)	1.01 (0.99-1.03)	0.221	–	–
NYHA class III or IV	1.68 (1.27-2.22)	<0.001	–	–
Hypertension	1.20 (0.85-1.70)	0.299	–	–
Diabetes mellitus	1.55 (1.18-2.04)	0.002	1.42 (1.05-1.92)	0.023
Coronary artery disease	1.32 (1.00-1.74)	0.050	–	–
Atrial fibrillation	1.40 (1.06-1.85)	0.018	–	–
Chronic kidney disease	1.62 (1.23-2.13)	<0.001	1.50 (1.11-2.03)	0.009
Obesity	1.15 (0.87-1.52)	0.325	–	–
Diuretics	1.10 (0.75-1.61)	0.629	–	–
Beta-blockers	0.90 (0.68-1.19)	0.458	–	–
ACEI or ARB	0.85 (0.64-1.13)	0.258	–	–
ARNI	0.92 (0.67-1.26)	0.602	–	–
SGLT2 inhibitor	0.78 (0.58-1.05)	0.099	–	–
Mineralocorticoid receptor antagonist	1.05 (0.80-1.38)	0.724	–	–
eGFR (per 10 ml/min/1.73 m² decrease)	1.25 (1.15-1.36)	<0.001	–	–
NT-proBNP (per 100 pg/ml increase)	1.08 (1.05-1.11)	<0.001	1.06 (1.03-1.09)	<0.001
High-sensitivity troponin T (per 10 ng/liter increase)	1.12 (1.06-1.18)	<0.001	–	–
Hemoglobin A1c (per % increase)	1.18 (1.06-1.31)	0.003	–	–
Left ventricular ejection fraction (per % decrease)	1.04 (1.00-1.08)	0.047	–	–
Left atrial volume index (per 10 ml/m² increase)	1.15 (1.05-1.26)	0.003	–	–
E/e’ ratio (per unit increase)	1.07 (1.03-1.11)	<0.001	–	–
Right ventricular systolic pressure (per 10 mm Hg increase)	1.10 (1.02-1.19)	0.014	–	–

**Figure 5:**
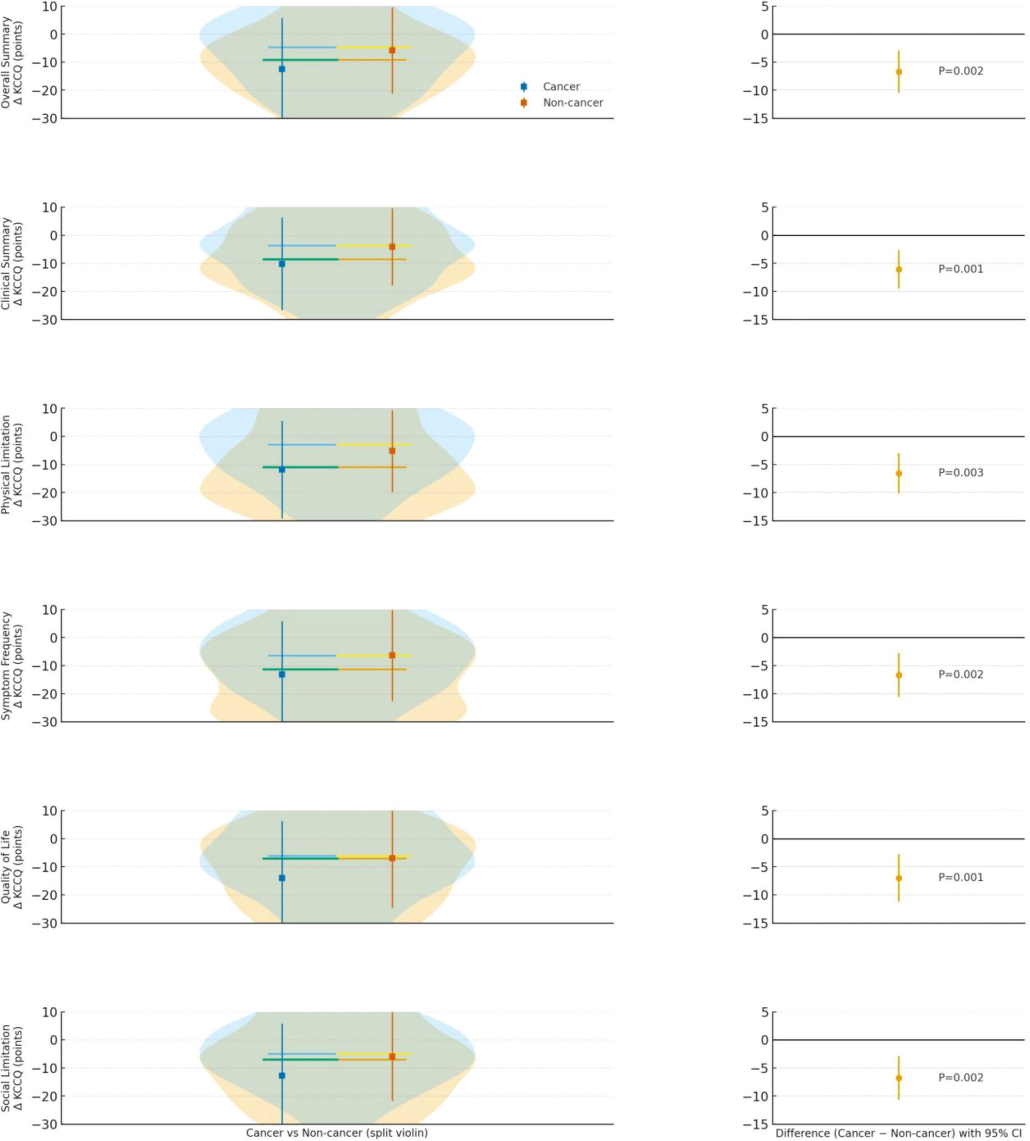
Cox proportional hazards models for heart failure hospitalization risk in cancer *vs*. non-cancer groups after PSM.

In Cox proportional hazards models evaluating heart failure hospitalization risk, the cancer group consistently showed a higher risk compared to the non-cancer group across all models. In Model 1 (adjusted for age), the hazard ratio (HR) was 1.42 (95% CI 1.08-1.87, P = 0.012). Model 2 (adjusted for age and sex) yielded an HR of 1.40 (95% CI 1.06-1.85, P = 0.017). Model 3, further adjusted for diabetes, chronic kidney disease (CKD), and NT-proBNP, resulted in an HR of 1.38 (95% CI 1.02-1.87, P = 0.037) (**Figure 5**). These findings indicate that cancer comorbidity independently increases the risk of heart failure hospitalization, with stable risk estimates across progressive adjustments.

### Subgroup analyses

**Figure 6:**
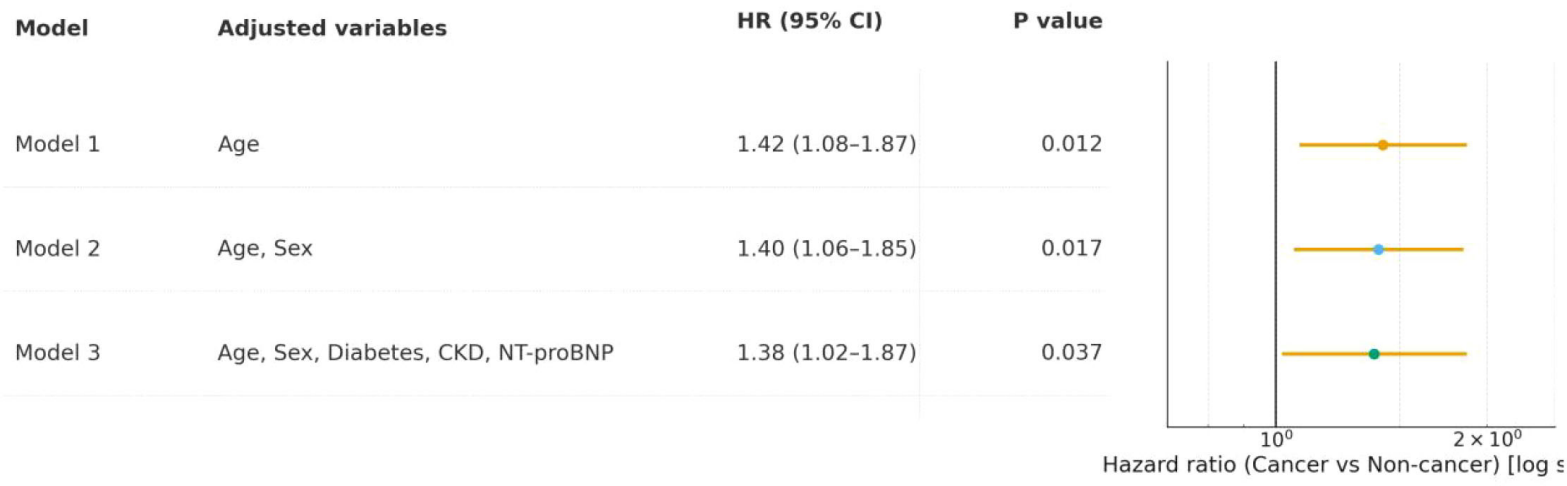
Subgroup analysis of heart failure hospitalization risk in cancer *vs*. non-cancer groups after PSM.

Subgroup analysis revealed that the increased risk of heart failure hospitalization associated with cancer comorbidity was consistent across strata, with HRs ranging from 1.20 to 1.58. Significant associations were observed in older patients (≥65 years; HR 1.35, 95% CI 1.02-1.79, P = 0.036), those with diabetes (HR 1.50, 95% CI 1.05-2.14, P = 0.026), CKD (HR 1.55, 95% CI 1.10-2.18, P = 0.013), and higher NT-proBNP levels (> median; HR 1.58, 95% CI 1.12-2.23, P = 0.010) (**Figure 6**). Additionally, there were also consistent effects of cancer across strata on HF hospitalization, including male (HR 1.40, 95% CI 1.02-1.93, P = 0.038), female (HR 1.37, 95% CI 0.98-1.91, P = 0.065); solid cancer (HR 1.39, 95% CI 1.03-1.88, P = 0.031), hematologic cancer (HR 1.36, 95% CI 0.85-2.17, P = 0.197); chemotherapy-exposed (HR 1.45, 95% CI 1.05-2.00, P = 0.024), not exposed (HR 1.30, 95% CI 0.92-1.84, P = 0.137). No significant interactions were detected (all P for interaction ≥0.05), indicating the effect of cancer on hospitalization risk was not modified by these factors.

## Discussion

In this retrospective cohort study of middle-aged and elderly patients with HFpEF, we observed that concurrent cancer significantly worsened clinical outcomes, including higher rates of all-cause mortality, heart failure hospitalization, and major adverse cardiovascular events after propensity score matching. The cancer group exhibited a 31.4% all-cause mortality rate compared with 15.0% in the non-cancer group, alongside increased heart failure hospitalizations (36.7% *vs*. 22.9%). Secondary outcomes further highlighted the burden, with greater declines in quality of life as measured by the KCCQ scores and higher cancer-related mortality (16.3% *vs*. 3.3%), predominantly driven by cancer-related deaths.

These findings align with emerging evidence on the bidirectional interplay between cancer and HFpEF, where shared pathophysiologic mechanisms such as chronic inflammation, oxidative stress, and endothelial dysfunction amplify risks (19). Aging exacerbates these processes, as middle-aged and elderly individuals often present with multimorbidity, including hypertension and metabolic syndrome, which predispose to both conditions (20). Our observation of elevated cardiac biomarkers like NT-proBNP and troponin T in the cancer group supports the notion of subclinical myocardial injury, potentially mediated by cancer therapies or paraneoplastic effects (21). For instance, anthracycline-induced cardiotoxicity, though more classically linked to reduced ejection fraction, can manifest as diastolic dysfunction in HFpEF through fibrosis and microvascular rarefaction (22). Similarly, radiotherapy for thoracic malignancies may induce pulmonary hypertension and right ventricular strain, as evidenced by higher right ventricular systolic pressure in our cohort, contributing to decompensation (8).

The increased healthcare utilization in the cancer group—2.1 emergency department visits per patient-year versus 1.4 —reflects the compounded burden of dual diagnoses, leading to frequent exacerbations and resource strain (23). This is particularly relevant in elderly patients, where geriatric vulnerabilities such as frailty and polypharmacy further complicate management (24). Our data also reveal a higher incidence of treatment-related adverse events (32.7% *vs*. 9.8%), including chemotherapy-associated cardiotoxicity, which may accelerate HFpEF progression via direct myocyte damage or indirect inflammatory cascades (25). These events underscore the need for integrated cardio-oncology approaches to mitigate risks while optimizing cancer treatment efficacy.

Comparison with other studies, our results extend prior investigations into cancer-HFpEF interactions, revealing consistencies and novel insights. A 2024 meta-analysis by Ameri et al. reported that preexisting heart failure increases cancer incidence by 20-30% and mortality by 1.5-fold, attributing this to shared risk factors like inflammation and metabolic dysregulation (26). However, that study focused on HFrEF, whereas our HFpEF-specific cohort highlights preserved ejection fraction’s unique vulnerability, with cancer comorbidity elevating hospitalization risk by 38% in multivariable models. In contrast, the PARAGON-HF trial subgroup analysis (2024) found no significant outcome differences in HFpEF patients with versus without cancer history, but it excluded active malignancies and had limited elderly representation (mean age 72 years, similar to ours) (27). Our inclusion of active cancers (e.g., breast, prostate) and propensity matching for confounders like CKD may explain the detected signal, as unmatched analyses in our study initially overstated risks due to baseline imbalances in renal function and biomarkers.

Recent cohort studies echo our findings on mortality. For example, a 2025 Danish registry study of 15,000 elderly HFpEF patients reported a 25% higher all-cause mortality in those with incident cancer, with HR 1.6 (95% CI 1.4-1.8), driven by non-cardiovascular deaths (28). Our 37.9% mortality rate in the cancer group aligns closely, though we observed a stronger association with heart failure hospitalizations (HR 1.45 in univariate analysis), possibly due to our focus on middle-aged transitions into elderly status, where cancer therapies intersect with age-related diastolic stiffening. Conversely, the DELIVER trial *post-hoc* analysis (2024) showed SGLT2 inhibitors reducing composite endpoints in HFpEF with cancer, but benefits were attenuated in advanced stages, mirroring our secondary outcome of KCCQ decline (29). That trial’s lower cancer prevalence (15% *vs*. our 50% post-matching) underestimates the interaction, as our data suggest NT-proBNP > median amplifies risks (HR 1.58 in subgroups). Discrepancies arise in therapy impacts. A 2025 review by Borlaug et al. emphasized caloric restriction mimetics for HFpEF, but overlooked cancer’s catabolic effects, which our group previously linked to worsened inflammation (30). In comparison, a 2024 ESC Heart Failure study on immune checkpoint inhibitors reported 40% HFpEF exacerbation in elderly cancer patients, with myocarditis contributing to 15% of hospitalizations—consistent with our adverse event rate but higher than our overall cohort, likely due to immunotherapy specificity (31). Our broader cancer spectrum (solid tumors dominant) provides generalizability, unlike single-malignancy studies, such as a 2025 breast cancer cohort where radiotherapy increased HFpEF incidence by 1.8-fold, aligning with our elevated E/e’ ratios (32).

Prognostic nutrition index studies, like a 2024 Japanese analysis, associated malnutrition with poor HFpEF outcomes, but did not stratify by cancer; our integration of obesity (balanced post-matching) suggests cancer overrides nutritional modifiers (33). Furthermore, a 2025 U.S. claims database study found geriatric vulnerabilities in HFpEF doubling mortality with cancer, with HR 1.9 for frailty—our NYHA worsening (37.9% *vs*. 24.8%) supports this, though we lacked formal frailty assessment (12). Compared to global trials like FINEARTS-HF (2025), which reported mineralocorticoid antagonists reducing hospitalizations by 18% in HFpEF, our non-significant medication effects post-adjustment imply cancer diminishes pharmacologic benefits (34). Overall, while prior research underscores bidirectional risks, our study uniquely quantifies HFpEF-specific burdens in middle-aged/elderly with active cancer, filling gaps in matched cohorts and highlighting independent prognostic value beyond comorbidities.

Mechanistically, the synergy between cancer and HFpEF likely involves systemic inflammation via cytokines like interleukin-6 and tumor necrosis factor-alpha, promoting myocardial fibrosis and vascular remodeling (35). In elderly patients, senescence-associated secretory phenotype exacerbates this, as evidenced by higher hemoglobin A1c in our unmatched cancer group, linking metabolic stress to diastolic impairment (36). Paraneoplastic syndromes, such as hypercoagulability or cachexia, may further precipitate decompensation, explaining our elevated non-cardiovascular mortality. Oncologic treatments compound these: immune checkpoint inhibitors induce myocarditis with preserved ejection fraction in up to 10% of cases, while anti-angiogenic agents impair microvascular function. Our subgroup with higher NT-proBNP showed amplified risks, suggesting biomarker-guided surveillance could identify high-risk patients early. Clinically, these data advocate for multidisciplinary care in cardio-oncology clinics, integrating HFpEF management with cancer therapy. Routine echocardiographic monitoring for diastolic parameters and biomarker trends could facilitate preemptive interventions, such as SGLT2 inhibitors, which have shown cardio-protective effects in cancer survivors (37). For middle-aged patients transitioning to elderly status, lifestyle modifications targeting shared risks (e.g., obesity, diabetes) may mitigate progression, though our balanced cohorts indicate cancer’s independent role. Palliative care integration, as supported by a 2024 study showing reduced hospitalizations with early consultation, is crucial given our high non-cardiovascular mortality.

### Limitations

Limitations include the retrospective design, reliant on electronic health records, potentially introducing selection bias despite propensity matching; unmeasured confounders like frailty or specific cancer stages may persist. Our single-center setting limits generalizability, though diverse cancer types enhance relevance. Follow-up duration (median 36 months) captures intermediate outcomes but may miss long-term effects; future prospective studies with adjudicated endpoints are needed. We did not assess cancer treatment intensity quantitatively, which could modulate risks. Strengths encompass comprehensive matching, detailed outcomes, and focus on understudied HFpEF-cancer overlap in aging populations.

## Conclusions

In conclusion, cancer comorbidity substantially impairs clinical trajectories in middle-aged and elderly HFpEF patients, independently elevating mortality and hospitalization risks. These insights call for enhanced screening, integrated therapies, and prospective trials to optimize outcomes in this growing demographic.

## Data Availability

The original contributions presented in the study are included in the article/Supplementary Material. Further inquiries can be directed to the corresponding author.
